# Efficacy of Combining Kegel Exercises with EMS-Based Pelvic Floor Muscle Electrostimulation in Postmenopausal Women with Involuntary Urinary Leakage

**DOI:** 10.3390/clinpract16050085

**Published:** 2026-04-29

**Authors:** Lucian Șerbănescu, Sebastian Mirea, Paris Ionescu, Ionuț Iorga, Traian-Virgiliu Surdu, Vadym Rotar, Stere Popescu, Elena Mocanu, Luana Alexandrescu, Cosmin Nișcoveanu, Radu-Andrei Baz

**Affiliations:** 1 Faculty of Medicine, Ovidius University of Constanta, 900470 Constanta, Romania or lucian_trocadero@yahoo.com (L.Ș.); paris.ionescu@365.univ-ovidius.ro (P.I.); ionut.iorga@365.univ-ovidius.ro (I.I.); traian@surdu.ro (T.-V.S.); rotarvadym.obst.gyn@gmail.com (V.R.); stere.popescu@365.univ-ovidius.ro (S.P.); alexandrescu_l@yahoo.com (L.A.); cosmin_niscoveanu@yahoo.com (C.N.); raduandreibaz@yahoo.com (R.-A.B.); 2 County Clinical Emergency Hospital “St. Apostle Andrew”, 900591 Constanta, Romania; sebastian.mirea@umft.ro; 3 Techirghiol Balneal and Rehabilitation Sanatorium, 906100 Constanta, Romania

**Keywords:** urinary incontinence, menopause, pelvic floor muscle training, electromagnetic stimulation, EMSella, Kegel exercises

## Abstract

Background/Objectives: Urinary incontinence (UI) is a frequent condition in postmenopausal women and is associated with a substantial negative impact on quality of life. Conservative management can include pelvic floor muscle training (PFMT) and high-intensity focused electromagnetic stimulation (HIFEM); however, data regarding the potential benefit of combining these modalities remain limited. This study aimed to evaluate whether the addition of a structured Kegel exercise program to EMSELLA-based electromagnetic stimulation is associated with enhanced clinical outcomes in postmenopausal women with urinary incontinence. Methods: This prospective comparative study included 99 postmenopausal women with stress, urgency, or mixed urinary incontinence and an International Consultation on Incontinence Questionnaire–Urinary Incontinence Short Form (ICIQ-UI SF) score ≥ 6. Participants received either EMSELLA therapy alone (Group A, n = 49) or EMSELLA combined with a standardized Kegel exercise program (Group B, n = 50) over a three-month period. Symptom severity was assessed at baseline and at three months using the ICIQ-UI SF. Between-group comparisons were performed using analysis of covariance, adjusting for baseline scores. Results: Both therapeutic approaches were associated with clinically meaningful improvement in urinary incontinence symptoms. After adjustment for baseline severity, lower follow-up ICIQ-UI SF scores, greater mean symptom reduction, and higher response rates were observed in the combined-therapy group. Across all menopausal-duration subgroups, outcomes consistently favored the association of EMSELLA therapy with Kegel exercises. No treatment-related adverse events were reported. Conclusions: The association of EMSELLA electromagnetic stimulation with a structured Kegel exercise program was associated with greater symptom improvement than electromagnetic stimulation alone, suggesting an additive therapeutic effect of voluntary pelvic floor muscle training. This combined conservative approach was well tolerated and may represent a useful management strategy for postmenopausal urinary incontinence.

## 1. Introduction

Urinary incontinence (UI), defined as the involuntary loss of urine, is a highly prevalent condition that disproportionately affects women and has substantial physical, psychological, and social consequences. Its impact on daily functioning, sexual health, and overall quality of life is well documented across epidemiological and clinical studies [1,2,3,4,5,6,7,8,9]. Major professional bodies, including the American College of Physicians and the American College of Obstetricians and Gynecologists, recognize several distinct subtypes of UI: stress urinary incontinence (SUI), urgency urinary incontinence (UUI), mixed urinary incontinence (MUI), overflow incontinence, and functional incontinence [2,3,6]. Accurate differentiation among these subtypes is essential, as management strategies vary considerably depending on underlying pathophysiology. Urge incontinence is characterized by involuntary urine loss triggered by a sudden and overwhelming need to urinate, often leading to distressing situations where individuals struggle to reach a restroom in time. This condition differs fundamentally from stress urinary incontinence (SUI), where leakage occurs during physical exertion, such as coughing or sneezing. However, it is not uncommon for individuals to experience both forms of incontinence simultaneously, resulting in a mixed urinary incontinence scenario that complicates management strategies [1,4,5].

The prevalence of UI increases with age and is strongly influenced by multiparity, vaginal delivery, obesity, hormonal changes associated with menopause, and comorbid medical conditions [2,3,4,8]. Despite its burden, UI remains frequently underreported, largely due to embarrassment, social stigma, or the misconception that urinary leakage is an inevitable component of aging [3,4]. Postmenopausal women represent a particularly vulnerable group, as estrogen deficiency and age-related changes in connective tissue, muscle tone, and bladder function contribute to both symptom onset and progression.

Conservative therapy is widely recommended as the first-line approach for most forms of non-neurogenic female UI. Among these, pelvic floor muscle training (PFMT)—commonly known as Kegel exercises—remains the cornerstone of behavioral treatment and is supported by robust evidence demonstrating improvement in symptoms, pelvic floor strength, and quality of life. Parallel to this, pelvic floor electrostimulation, including high-intensity focused electromagnetic stimulation (HIFEM) devices such as EMSella, has gained increasing attention for its ability to induce supramaximal contractions and promote neuromuscular reactivation of the pelvic floor.

While both PFMT and HIFEM-based electrostimulation independently demonstrate therapeutic benefits, the extent to which their combination produces superior outcomes in postmenopausal women is not fully established. Given the multifactorial pathophysiology of UI in this population—characterized by muscular atrophy, connective tissue weakening, and reduced neuromuscular control—it is plausible that a multimodal approach could yield additive or synergistic improvements.

Therefore, the present study aims to evaluate the efficacy of combining Kegel exercises with EMSella-based pelvic floor electrostimulation, compared with electrostimulation alone, in postmenopausal women experiencing involuntary urinary leakage. By using the validated International Consultation on Incontinence Questionnaire—Urinary Incontinence Short Form (ICIQ-UI SF) to assess outcomes at baseline and three months, this study seeks to determine whether the addition of PFMT provides a clinically meaningful enhancement in symptom reduction.

## 2. Materials and Methods

### 2.1. Study Design and Duration

This prospective comparative study was conducted over a period of 3 months and included postmenopausal women diagnosed with involuntary urinary leakage. All patients included in this study were evaluated by the same multidisciplinary medical team consisting of gynecologists, urologists, and surgeons. Although it is not always possible to make a clear clinical classification of the type of involuntary urine loss, especially in mixed cases, an attempt was made to include patients with SUI in the study as patients with MUI. We mention that no patient accepted into the study received drug therapy for involuntary urine loss during the study. Cases were allocated randomly into two intervention groups for each clinically classified SUI and MUI group.

• Group A: EMSELLA therapy alone (n = 49).
• Group B: EMSELLA therapy combined with Kegel exercises (n = 50).

To analyze the influence of the menopausal duration on therapeutic response, each group was stratified into three subgroups according to time since the onset of menopause:

• Subgroup 1: menopause for approximately 5 years.
• Subgroup 2: menopause between 5 and 10 years.
• Subgroup 3: menopause between 10 and 15 years.

### 2.2. Inclusion and Exclusion Criteria

Inclusion criteria:

• Postmenopausal women (≥12 months of amenorrhea);
• Age 45–70 years;
• Presence of involuntary urinary leakage (stress or mixed type);
• ICIQ-UI Short Form score ≥ 6;
• Ability to understand and correctly perform pelvic floor muscle exercises (if assigned to Group B);
• No hormone replacement therapy in the past 12 months (systemic, topical, or localized)—mandatory criterion or drug therapy for involuntary urine loss during the study.

Exclusion criteria:

• Active urinary tract infection or genital infection;
• History of pelvic malignancy;
• Previous pelvic floor surgery or mid-urethral sling;
• Use of topical or systemic estrogen therapy within the last year;
• Neurological conditions affecting bladder or pelvic floor function;
• Presence of pacemakers, metallic implants or contraindications to electromagnetic stimulation;
• Cognitive impairment limiting exercise compliance;
• Pelvic organ prolapse stage ≥ II (POP-Q).

### 2.3. Treatment Protocol

#### 2.3.1. EMSELLA Electromagnetic Stimulation Protocol

All participants underwent therapy with an EMSELLA^®^ (BTL Industries, Prague, Czech Republic) device, following the international protocol for urinary incontinence management:

• Session frequency: 2 sessions per week, three consecutive weeks.
• Total number of sessions: 6 sessions.
• Duration: 28 min per session.
• Stimulation intensity: gradually increased to the maximum tolerated level, typically ≥80% of device output.
• Position: seated upright, pelvis aligned with the electromagnetic field focus.
• Therapeutic goal: induction of supramaximal pelvic floor contractions (≈11,000 contractions/session).

Compliance was documented at each visit.

#### 2.3.2. Kegel Exercise Protocol (Applied ONLY to Group B)

The Kegel training was standardized and instructed by a specialist physiotherapist. The protocol strictly followed the structure shown in the reference image:

• Duration: 3 months.
• Frequency: Twice daily.
• Patient position: Supine, with thighs flexed at a 90-degree angle to the abdomen.
• Session length: 15 min.

Month 1:

• Pelvic floor contraction 3 s.
• Relaxation 3 s.
• Continuous repetition throughout the 15 min session.
• Two sessions per day.

Months 2 and 3:

• Pelvic floor contraction 5 s.
• Relaxation 5 s.
• Continuous repetition for 15 min.
• Two sessions per day.

Patients were instructed to avoid abdominal, gluteal, or thigh muscle co-contraction and to focus on lifting and tightening the pelvic floor.

Adherence was monitored through a daily exercise log.

Outcome Measurements

All patients were evaluated at baseline and again at 3 months using the International Consultation on Incontinence Questionnaire—Urinary Incontinence Short Form (ICIQ-UI SF), a validated and widely used tool for quantifying symptom severity and quality-of-life impact.

No additional treatments, pharmacologic or physical, were permitted during the study period.

All patients included in the study were reevaluated three months after starting therapy. It should be emphasized that patients who underwent only EMSELLA therapy for three weeks did not follow any other therapy until the final evaluation, while those who combined it with PFMT started Kegel exercises on the first day of therapy, practically combining them with EMSELLA during the first three weeks, and then continued daily only with PFMT for three months according to the protocol described.

## 3. Results

Baseline demographic and clinical characteristics are summarized in Table 1. No statistically significant differences were observed between the two groups with respect to age, body mass index (BMI), parity, duration of menopause, type of urinary incontinence, or baseline ICIQ-UI SF score (all *p* > 0.05).

The mean age was 58.2 ± 6.1 years in Group A and 57.9 ± 6.4 years in Group B (*p* = 0.81). The mean BMI was 27.6 ± 3.4 kg/m^2^ and 27.9 ± 3.7 kg/m^2^, respectively (*p* = 0.67). Parity ≥2 was recorded in 33/49 patients (67.3%) in Group A and 35/50 patients (70.0%) in Group B (*p* = 0.77).

With regard to menopausal duration, 23 patients in Group A and 24 patients in Group B had been menopausal for approximately 5 years, 16 patients in each group between 5 and 10 years, and 10 patients in each group between 10 and 15 years (*p* = 0.93).

Stress urinary incontinence was the most common subtype, affecting 38 patients in Group A and 38 patients in Group B, and mixed urinary incontinence (11 vs. 12 patients, respectively). The distribution of incontinence subtypes did not differ significantly between groups (*p* = 0.98).

Baseline ICIQ-UI SF scores were comparable between groups (Group A: 12.4 ± 2.1; Group B: 12.6 ± 2.3; *p* = 0.72).

### 3.1. Primary Outcome: ICIQ-UI SF Score at 3 Months (ANCOVA)

The primary efficacy analysis was performed using analysis of covariance (ANCOVA), with the ICIQ-UI SF score at 3 months as the dependent variable, treatment group as the fixed factor, and baseline ICIQ-UI SF score as a covariate (Table 2).

At 3 months, the observed mean ICIQ-UI SF score was 9.4 ± 2.6 in Group A and 8.6 ± 2.5 in Group B. While the unadjusted between-group difference at follow-up did not reach statistical significance (*p* = 0.10), ANCOVA demonstrated a significant adjusted treatment effect.

After adjustment for baseline severity, Group B exhibited a significantly lower ICIQ-UI SF score compared with Group A, with an adjusted mean difference (Group B − Group A) of −0.68 points (95% confidence interval −1.26 to −0.10; *p* = 0.024). Baseline ICIQ-UI SF score was a significant predictor of the 3-month score (β ≈ 0.54, *p* < 0.001).

For descriptive purposes, the mean reduction from baseline was 3.0 ± 2.4 points in Group A and 4.0 ± 2.6 points in Group B.

**Table 2:** ICIQ-UI SF outcomes and primary ANCOVA analysis.

Outcome	Group A (n = 49)	Group B (n = 50)	*p*-Value
Baseline ICIQ-UI SF, mean ± SD	12.4 ± 2.1	12.6 ± 2.3	0.72
3-month ICIQ-UI SF, mean ± SD	9.4 ± 2.6	8.6 ± 2.5	0.10
Mean change (ΔICIQ), mean ± SD	3.0 ± 2.4	4.0 ± 2.6	**0.04**
ANCOVA-adjusted difference (B–A)	—	−0.68 (95% CI −1.26 to −0.10)	0.024
Baseline covariate (β)	—	≈0.54	<0.001

### 3.2. Subgroup Analysis by Duration of Menopause

Changes in ICIQ-UI SF score stratified by duration of menopause are presented in Table 3. In both treatment groups, symptom improvement decreased with increasing menopausal duration. Across all strata, numerical improvements consistently favored the combined EMSELLA + Kegel intervention, although between-group differences within individual subgroups did not reach statistical significance.

### 3.3. Clinically Meaningful Improvement, Patient Satisfaction, and Safety

Clinically meaningful improvement, defined as a reduction of at least 3 points in the ICIQ-UI SF score or a final score ≤ 2, was achieved by 28/49 patients (57.1%) in Group A and 38/50 patients (76.0%) in Group B (*p* = 0.047) (Table 4).

The odds of achieving a clinically meaningful response were higher in Group B (odds ratio 2.38, 95% CI 1.00–5.62). After adjustment for age, BMI, baseline ICIQ-UI SF score, and menopausal duration, the treatment group remained independently associated with response (adjusted OR ≈ 2.2, *p* ≈ 0.048).

Patient-reported satisfaction at 3 months was significantly higher in Group B (3.9 ± 0.8) compared with Group A (3.5 ± 0.9, *p* = 0.02). No treatment-related adverse events were reported in either group. Adherence to the Kegel exercise protocol in the first week exceeded 85% of prescribed sessions in 43/50 patients (86.0%) and, after this, in 50/50 patients (100%). This percentage of 14% (7/50) of the patients stated that just in the first week of Kegel exercises, it was difficult for them to perform these exercises in 2 sessions of 15 min; instead, they performed 30 min of Kegel exercises per day, but fragmented into several sessions (3–4 sessions) in the first week period.

**Table 4:** Clinical response, satisfaction, and safety outcomes.

Outcome	Group A (n = 49)	Group B (n = 50)	*p*-Value
Responders, n (%)	28 (57.1)	38 (76.0)	**0.047**
Non-responders, n (%)	21 (42.9)	12 (24.0)	
Odds ratio for response (95% CI)	—	2.38 (1.00–5.62)	—
Satisfaction score (Likert 1–5), mean ± SD	3.5 ± 0.9	3.9 ± 0.8	0.02
Adverse events	0	0	—
adherence to Kegel protocol	—	50 (100%)	—

## 4. Discussion

### 4.1. Principal Findings

The present study demonstrates that both therapeutic approaches—high-intensity focused electromagnetic pelvic floor stimulation (EMSELLA) alone and its combination with a structured Kegel exercise protocol—resulted in significant improvement in urinary incontinence severity over a 3-month treatment period in postmenopausal women. However, the magnitude of improvement was greater in the combined-therapy group.

Women who received EMSELLA plus the standardized Kegel regimen achieved a significantly larger reduction in ICIQ-UI SF scores compared to those treated with EMSELLA alone, and a higher proportion reached clinically meaningful improvement thresholds. These findings support the hypothesis that pelvic floor muscle activation through voluntary contractions provides an additive benefit when layered on to neuromuscular stimulation.

Importantly, the superiority of the combined approach was consistent across all menopausal duration subgroups (5, 10, and 15 years), suggesting that the synergistic effect of active and passive pelvic floor training is maintained regardless of the time elapsed since menopause.

### 4.2. Comparison with Existing Literature

Pelvic floor muscle training (PFMT, Kegel exercises) is an effective intervention for reducing urinary incontinence symptoms in postmenopausal women, with significant improvement rates demonstrated across meta-analyses and randomized controlled trials [10,11,12,13]. Recent literature supports PFMT as a first-line therapy, reporting cure or improvement rates of 56–74% for stress urinary incontinence and a 67–92% likelihood of clinically meaningful benefit compared with control groups [1,4,10,12,13].

Supervised PFMT programs—typically involving 8–30 contractions per day over a minimum of three months—are associated with superior outcomes compared with unsupervised regimens, while individual and group-based supervision offer comparable effectiveness [1,14,15,16]. PFMT is considered safe, with rare and minor adverse effects (such as vaginal discomfort or spotting), and is effective across all types of urinary incontinence; however, evidence regarding long-term efficacy (beyond one year) and optimal regimens for specific subgroups (e.g., women with genitourinary syndrome of menopause) remains limited [10,11,15].

Major professional bodies, including the American Academy of Family Physicians and international consensus groups, recommend PFMT as the first-line therapeutic option, noting its minimal risk profile and low associated costs. The commonly advised dosage consists of three sets of 8–10 contractions daily, accompanied by weekly supervision to maximize outcomes [1,4].

Electrical Muscle Stimulation, as a generic term, technologies—including EMS (Electrical Muscle Stimulation), NMES (Neuromuscular Electrical Stimulation), FES (Functional Electrical Stimulation), and TENS (Transcutaneous Electrical Nerve Stimulation)—have long been incorporated into both clinical and research settings as modalities for enhancing muscle function, mitigating disuse atrophy, and reducing pain. Evidence supporting their efficacy spans multiple populations, from healthy adults to older individuals, critically ill patients, and those recovering from neurological injury. According to the 2024 Department of Veterans Affairs guideline, FES, NMES, and TENS improve motor outcomes in both upper and lower limbs, contributing to increased gait speed, enhanced motor performance, and improved functional scores such as the Barthel Index and Fugl–Meyer Assessment in patients with stroke; these conclusions are supported by meta-analyses and randomized controlled trials, despite considerable heterogeneity across stimulation protocols [17]. In healthy populations, EMS reliably increases muscular strength, although consistent improvements in physical function or body composition have not been demonstrated. The absence of standardized stimulation parameters remains a major limitation, although intensities ≥50% of maximal voluntary contraction appear most conducive to strength adaptation. Within athletic contexts, EMS may serve as an adjunct to resistance training but does not substitute for voluntary exercise [18,19].

In critically ill individuals, EMS has been shown to attenuate upper and lower limb muscle atrophy and may contribute to reduced hospitalization duration without significantly altering the incidence of ICU-acquired weakness [20]. Among elderly patients and those with chronic diseases, EMS demonstrates additional potential in preventing sarcopenia, improving muscular performance, supporting glycemic control, and facilitating orthopedic and cardiac rehabilitation [21,22]. Collectively, the literature positions EMS as a validated strategy for increasing muscle strength, preventing atrophy, and supporting functional recovery across diverse clinical populations, while underscoring the persistent need for standardized application protocols [17,18,19,20,21,22].

The safety profile of EMS is generally favorable. The most frequently reported adverse effects—including local discomfort, pain, tingling, burning sensations, muscle fatigue, and delayed-onset muscle soreness—are typically mild and transient across both healthy and clinical cohorts [23,24]. More intense protocols may induce muscle microinjury, reflected by elevations in creatine kinase, reduced muscle strength, and post-exercise soreness, paralleling the effects of eccentric voluntary contractions but with limited tissue involvement [25,26]. Perceived discomfort is influenced by stimulation parameters and electrode configuration, with lower discomfort observed during asynchronous stimulation and when larger electrodes are used [23]. Although rare, severe complications such as rhabdomyolysis and acute compartment syndrome have been documented, predominantly in individuals with underlying chronic illnesses or myopathies, highlighting the importance of careful patient selection and monitoring—including renal function and electrolyte balance—particularly in vulnerable populations [27]. Minor reactions such as pruritus, headache, general fatigue, or superficial skin burns have also been reported [2], while clinically relevant cardiac arrhythmias have not been associated with standard therapeutic intensities [28]. Overall, EMS-related adverse events remain uncommon and typically mild, yet the possibility of severe complications in high-risk individuals reinforces the necessity of rigorous clinical oversight [23,24,25,26,27].

Evidence for combining pelvic floor muscle training (PFMT) with electrical stimulation (ES) is limited and low quality, showing no clear benefit over either therapy alone [29,30,31,32,33]. Cochrane data confirm no significant added improvement when PFMT is added to ES [29].

Consistent with prior evidence showing that Kegel exercises enhance treatment efficacy across various urologic and gynecologic conditions—including urge urinary incontinence and mixed urinary incontinence—the present findings further support the added therapeutic value of integrating pelvic floor muscle training with established interventions for improved continence outcomes [34,35].

This study addresses a critical knowledge gap by providing the first comparative evidence that combining high-intensity electromagnetic stimulation with a standardized Kegel protocol yields superior improvements in urinary incontinence severity among postmenopausal women, a population for whom data on integrated pelvic floor rehabilitation strategies remain limited.

Recent findings in vulvovaginal atrophy similarly demonstrate that multimodal approaches—such as estriol plus Kegel exercises—provide superior clinical outcomes versus single-therapy regimens, reinforcing the rationale for combined pelvic floor treatments in postmenopause [36,37].

### 4.3. Physiological Rationale

The enhanced therapeutic effect observed with the combined EMSELLA + Kegel protocol is physiologically plausible, as the two interventions target complementary mechanisms of pelvic floor strengthening. High-intensity focused electromagnetic stimulation induces rapid, supramaximal contractions that cannot be achieved voluntarily, promoting neuromuscular recruitment and hypertrophic adaptation of deep pelvic floor fibers. In contrast, structured Kegel exercises reinforce voluntary motor control, endurance, and coordination of the pelvic musculature. When applied together, these modalities likely generate both neural and structural improvements—optimizing muscle activation patterns, increasing contraction efficiency, and enhancing urethral support—thereby producing a more pronounced reduction in urinary incontinence severity than either method alone.

Moreover, recent evidence demonstrating tissue-level adaptations following short-course balneotherapeutic interventions—such as increased capillary caliber, angiogenesis, and sustained histological changes in dermal and muscular structures after only two weeks of treatment—underscores the broader principle that non-pharmacological physical therapies can induce measurable biological remodeling, reinforcing the plausibility of the enhanced neuromuscular response observed with our combined ES + Kegel protocol [38].

### 4.4. Clinical Implications

These findings underscore the clinical value of combining ES with a structured Kegel protocol in postmenopausal women, demonstrating that a dual-modality approach yields greater functional improvement than electrostimulation alone. The integration of voluntary pelvic floor activation with high-intensity focused stimulation appears to enhance neuromuscular adaptation, accelerate symptom reduction, and increase the likelihood of clinically meaningful improvement at three months. Taken together, the data support the premise that multimodal pelvic floor rehabilitation offers a superior therapeutic strategy, particularly in populations with age-related muscle weakness and declining estrogen levels, where single-intervention approaches may be insufficient.

Our results reinforce contemporary evidence that multimodal, individualized approaches provide the most effective therapeutic benefit for urinary incontinence in menopausal women [39,40,41].

The microbiome of the urinary bladder and intestinal tract plays a key role in maintaining genitourinary health, good functionality, and the prevention of possible infections with pathogenic germs. Alteration of this microbiome can lead to the appearance of so-called subacute urinary tract infections, which can potentiate involuntary urine losses, especially in menopausal women, in whom hypoestrogenism substantially modifies the genitourinary microbiome [42].

Urogynecological surgical procedures in women over 80 years of age represent a great challenge for the operating teams due to the associated chronic pathology [43]. A conservative approach in these cases may represent the only therapeutic solution.

### 4.5. Strengths and Limitations

The primary strengths of this study lie in its rigorous comparative design, which evaluated two clearly defined intervention groups under standardized therapeutic conditions. The use of the internationally validated ICIQ-UI SF ensured high methodological reliability and allowed for objective assessment of symptom evolution. Both the EMSELLA protocol and the structured Kegel exercise program were uniformly applied, minimizing variability in treatment exposure. Additionally, the exceptionally high adherence rate among participants reinforces the internal validity of the findings and supports the robustness of the observed clinical outcomes.

This study has several limitations that should be acknowledged. The follow-up period was relatively short (3 months), which may not fully capture long-term therapeutic durability. Adherence to the Kegel protocol was self-reported, introducing a potential compliance bias. Additionally, the absence of a placebo or sham-stimulation group limits the ability to isolate the specific contribution of each intervention component. Finally, objective pelvic floor measurements such as perineometry or EMG (Electromyography) were not included, which may have strengthened the physiological interpretation of the findings. Nevertheless, these limitations do not undermine the clinical relevance of the observed improvements.

Future research should prioritize long-term follow-up at 6–12 months to determine the durability of therapeutic gains, integrate objective physiological markers such as EMG (Electromyography) and manometry to refine mechanistic understanding, and employ multicenter randomized controlled designs to enhance generalizability.

The combined use of high-intensity pelvic floor electromagnetic stimulation and a structured Kegel exercise protocol produced significantly greater improvements in urinary incontinence severity than EMSELLA alone. These benefits were consistent across all menopausal duration subgroups, supporting the integration of voluntary pelvic floor training with electromagnetic therapy as a more effective conservative strategy for postmenopausal urinary incontinence.

The fact that the duration of the Kegel exercise sequences was 15 min was, on the one hand, an advantage because this intense, sustained effort led to better control over the sphincter muscles, and on the other hand, it could also represent an impediment for the patients. In order to overcome this impediment and convince the patients to strictly follow the Kegel exercise program, our protocol was lighter in the first month (3 s contraction, 3 s relaxation) and more intense in months 2 and 3 (5 s contraction, 5 s relaxation).

## 5. Conclusions

In this prospective study both EMSELLA monotherapy and the combined EMSELLA + Kegel protocol resulted in meaningful clinical improvement after three months. However, the addition of a structured pelvic floor exercise regimen produced significantly greater reductions in ICIQ-UI SF scores, higher rates of clinically relevant improvement, and superior outcomes across all menopausal-duration subgroups. These findings support the synergistic effect of integrating voluntary pelvic floor muscle activation with high-intensity focused electromagnetic stimulation, particularly in populations with age-related declines in neuromuscular responsiveness.

The combined approach was well tolerated, with high adherence and no adverse events, reinforcing its suitability as a safe, conservative first-line therapy. Future research should explore longer-term follow-up, optimization of exercise parameters, and the potential role of individualized protocols based on menopausal duration, symptom phenotype, and pelvic floor functional status.

## Figures and Tables

**Table 1:** Baseline demographic and clinical characteristics of the study population.

Characteristic	Group A (EMSella) n = 49	Group B (EMSella + Kegel) n = 50	*p*-Value
Age (years), mean ± SD	58.2 ± 6.1	57.9 ± 6.4	0.81
BMI (kg/m^2^), mean ± SD	27.6 ± 3.4	27.9 ± 3.7	0.67
Parity ≥ 2, n (%)	33 (67.3)	35 (70.0)	0.77
Duration of menopause, n (%)			0.93
– ~5 years	23 (46.9)	24 (48.0)	
– ~10 years	16 (32.7)	16 (32.0)	
– ~15 years	10 (20.4)	10 (20.0)	
Type of urinary incontinence, n (%)			0.98
– Stress UI	38 (77.5)	38 (76.0)	
– Mixed UI	11 (22.4)	12 (24.0)	
Baseline ICIQ-UI SF, mean ± SD	12.4 ± 2.1	12.6 ± 2.3	0.72

**Table 3:** Change in ICIQ-UI SF score according to menopausal duration.

Duration of Menopause	Group A (EMSELLA)	Group B (EMSELLA + Kegel)	*p*-Value
~5 years	n = 23	n = 24	
Mean ΔICIQ ± SD	3.4 ± 2.3	4.3 ± 2.5	0.11
~10 years	n = 16	n = 16	
Mean ΔICIQ ± SD	3.0 ± 2.4	3.9 ± 2.6	0.18
~15 years	n = 10	n = 10	
Mean ΔICIQ ± SD	2.5 ± 2.5	3.3 ± 2.6	0.31

## Data Availability

The original contributions presented in this study are included in the article. Further inquiries can be directed to the corresponding author.

## Abbreviations

The following abbreviations are used in this manuscript:

UI	Urinary incontinence
SUI	Stress urinary incontinence
UUI	Urgency urinary incontinence
MUI	Mixed urinary incontinence
PFMT	Pelvic floor muscle training
HIFEM	High-intensity focused electromagnetic stimulation
ICIQ-UI SF	International Consultation on Incontinence Questionnaire—Urinary Incontinence Short Form
POP-Q	Pelvic organ prolapsed
BMI	Body mass index
EMS	Electrical Muscle Stimulation
NMES	Neuromuscular Electrical Stimulation
FES	Functional Electrical Stimulation
TENS	Transcutaneous Electrical Nerve Stimulation
ES	Electrical stimulation
EMG	Electromyography
